# Knowledge and attitude toward oral health behavior of overseas students during the COVID-19 pandemic

**DOI:** 10.1186/s12903-023-03420-1

**Published:** 2023-10-28

**Authors:** Isi Susanti, Pagaporn Pantuwadee Pisarnturakit, Neeracha Sanchavanakit

**Affiliations:** 1 Oral Biology Program, Faculty of Dentistry, Chulalongkorn University, Bangkok, 10330 Thailand; 2 Department of Community Dentistry, Faculty of Dentistry, Chulalongkorn University, Bangkok, 10330 Thailand; 3 Department of Anatomy, Faculty of Dentistry, Chulalongkorn University, Bangkok, 10330 Thailand

**Keywords:** Attitude, Behaviors, COVID-19, Knowledge, Oral healthcare, Overseas university students

## Abstract

**Background:**

The COVID-19 pandemic has impacted overseas students, including their oral health. Due to movement restrictions, limited living allowances, dental treatment costs, and health insurance fees, overseas students might be more concerned about their oral health. The objective of the present study was to determine the association of knowledge and attitude toward oral healthcare behavior of overseas university students staying in Thailand between January 2020 to July 2022 and explore the experiences of their oral health problems.

**Methods:**

A cross-sectional study was conducted using an online survey in English operated through the Google platform by convenience sampling among overseas Chulalongkorn University students. A newly developed self-administered questionnaire on knowledge and attitude toward oral health-related behavior and experiences in oral health problems was completed voluntarily. Descriptive statistics, Chi-square test, t-test, ANOVA, and Pearson correlations were employed using IBM SPSS version 29.

**Results:**

Of 311 overseas students, 55.6% were male. The average age of students was 27.5 ± 4.5 years. 68.81% of students were from ASEAN countries, and 73.31% studied in non-health science programs. The study fields, health and non-health sciences, were associated with knowledge score (p < 0.001) and attitude score (p = 0.004), whereas the type of health insurance had an association with behavior score (p = 0.014) and the student’s perspective about dental visits (p = 0.014). Three hundred fifty-nine cases of oral health problems were experienced by 47.3% of overseas students. These problems consisted primarily of tooth hypersensitivity (21.2%), gingivitis (15.3%), caries (14%), cracked or broken tooth (10%), severe toothache (9%), fallen out filling (8%), and wisdom tooth pain (7.8%). There was an association between oral healthcare behavior and oral health problems (p < 0.001), and a negative correlation was found between behavior score and the number of oral health problems (p < 0.001, r=-0.204).

**Conclusion:**

The oral healthcare habits of overseas university students correlated positively with knowledge and attitude. A negative correlation was observed between behavior and the number of oral health problems. Furthermore, studying in health science programs impacted students’ knowledge and attitude toward oral health, while dental treatment coverage insurance affected decisions for dental visits.

## Background

The World Health Organization (WHO) declared the outbreak of COVID-19 as a pandemic in March 2020. COVID-19 cases dramatically increased worldwide [1], destroying the economy and healthcare system [2]. In addition, all academic institutes were closed and turned to online teaching and learning modes following government rules and regulations [3]. Thailand witnessed several waves of COVID-19 outbreaks, and the government locked down the main cities or regions several times to minimize and control the spread of the disease [4, 5]. City lockdown and studying from home affected students’ lifestyles, mental health, food choices, and dietary habits [6]. There were both negative and positive impacts of the COVID-19 lockdown on dietary intakes around the world [7]. Negative diet patterns were linked to poor lifestyle outcomes such as weight gain, mental health concerns, and lack of physical exercise. These changed lifestyles could affect the health status in the short and long term if sustained [8].

Oral prevention and treatment are parts of the healthcare system. The dental practice has been rated in the most incredible risk categories regarding viral transmission. Almost all dental procedures generate a high volume of aerosols, and the close distance between the patient and the dentist increases the potential for transmission [9]. The American Dental Association (ADA) recommended postponing all dental treatments except those considered urgent or emergency [10]. Thus, the routine checkup and non-emergency treatments for any oral problem were canceled. During the pandemic, increases in the frequency of daily tooth brushing brought about incidences of tooth hypersensitivity and dislodged fillings [11].

Currently, the proportion of overseas students is gradually increasing in Thailand [12]. Being away from their home countries, overseas students have concerns for their health, including oral health, as it probably impacts the quality of study and expenses for living. Health insurance must be applied as the requirement for enrollment; however, affordable health insurance is generally not full coverage and probably none for dental treatment [13]. Oral healthcare behavior and consumption lifestyles, along with students’ cultural background and abroad experiences, influence awareness of individual health status during the normal situation. However, due to the COVID-19 pandemic, overseas students had to stay in the countries where they were because of the global lockdown. Also, they enforced social distance measures by studying from home via online classes because of the local lockdown [14]. Therefore, the pandemic could impact overseas students in multiple aspects, such as physical, psychological, and emotional well-being [15], including oral health. In this situation, overseas students need to be more concerned about their oral health.

Although reports on the association between knowledge and attitude toward oral healthcare behavior during the COVID-19 pandemic have been published for different populations [16], studies on overseas university students during COVID-19 restrictions have not been performed. We hypothesized that proper oral healthcare behavior of overseas university students might positively associate with their knowledge and attitude, and the COVID-19 pandemic might enhance the proper behavior. Dental caries and gingivitis are considered global shared major oral health problems with high prevalence in all age ranges [17]. Thus, the objective of the present study was to determine the association of knowledge and attitude toward oral healthcare behavior of overseas university students who remained in Thailand and to determine oral health problems during the COVID-19 pandemic.

## Methods

### Study Design and participants

A cross-sectional study was conducted using convenience sampling with overseas students at Chulalongkorn University. The inclusion criteria were overseas students studying at Chulalongkorn University over 18 years old and staying in Thailand for any period between January 2020 and July 2022. The period was from January 2020, when the COVID-19 outbreak was officially announced in Thailand, until the end of the academic year 2021 in July 2022, when online classes were terminated at Chulalongkorn University. This timeframe helped the target population to remember the situation and reduce the recall bias. The exclusion criteria was overseas students studying in dental professional programs. The sample size was calculated based on 826 students, a total number of the overseas student population of Chulalongkorn University in the academic year 2021 (August 2021-July 2022), using the Taro Yamane Formula as follows [18].

$$n=\frac{N}{1+N{e}^{2}}$$

$$n=\frac{826}{1+826 {\left(0.05\right)}^{2}}$$

$$n=270$$

Where: *n* = Sample size, *N* = Total population, *e* = Standard error (5%).

By the calculation, the minimum sample size was 270. A 10% compensation was added for data missing by incomplete data. Hence, the minimum sample size was 297. The overseas students’ countries were categorized as ASEAN (The Association of Southeast Asian Nations) and non-ASEAN countries.

### Ethics approval

The study was performed following the Declaration of Helsinki and approved by the Human Research Ethics Committee of the Faculty of Dentistry, Chulalongkorn University (study code HREC-DCU 2022 − 110). The questionnaire was completed voluntarily. The participants were informed that they could withdraw from the research or terminate the questionnaire at any time. Completing and submitting this questionnaire was regarded as consent to participate.

### Questionnaire and data collection

The data was collected using a newly developed self-administered questionnaire on knowledge and attitudes toward oral health-related behavior. Extensive literature reviews on oral health and related topics relevant to the study were conducted and used to develop the key items needed for the questionnaire’s domains. The questionnaire was initially developed in English. Three experts in oral healthcare evaluated the questionnaire for validity using IOC (Item-Objective Congruence Index). They selected items for precision in questions, accuracy in knowledge, and interpretability in attitude and behavior. Additionally, the experts assessed the content validity of initial questionnaire questions. The reliability of the questionnaire was tested by 30 overseas students from other universities in Bangkok with the same characteristics as the target participants. A Cronbach’s alpha coefficient was calculated to determine the internal consistency (IC) of the questionnaire’s items which was ≥ 0.7 in the present study.

The questionnaire was divided into five sections: (1) sociodemographic characteristics, (2) 13 questions to assess knowledge related to oral diseases, habits, and oral healthcare, which answered with “yes”, “no” and “I do not know”, (3) 14 statements for attitudes towards oral diseases, habits, and oral healthcare were measured by a 5-point Likert scale “strongly agree”, “agree”, “neither agree nor disagree”, “disagree” and “strongly disagree”. The results were categorized into positive and negative attitudes. For interpretation as the positive attitude, the cut-off point on the positive statements was between 4 (agree) and 3 (neither agree nor disagree), while the cut-off point on the negative statements was between 3 (neither agree nor disagree) and 2 (disagree), (4) 11 questions with multiple choices for behaviors related to oral health during the COVID-19 pandemic and also the overview of “increased”, “decreased” or “no change” behavior measured by the personal perception of respondents being compared to that before the pandemic, and (5) experiences in oral health problems during the pandemic with multiple answers.

Data collection was carried out between January and February 2023. An online survey operated through Google form. We conducted sampling using the convenience strategy [19]. Through the overseas student networks of Chulalongkorn University, a recruitment link was distributed to groups in Line, Facebook, or other social media types. Then the individuals in this group further distributed to the others who could participate in the survey voluntarily. This process continued until the sample size met the requirements. Participants were excluded from the data analysis if they did not meet the inclusion and exclusion criteria.

### Statistical analysis

All data analysis was conducted using IBM SPSS Statistics version 29. Descriptive statistics were performed to analyze sociodemographic data, knowledge score, attitudes score, and behavior score. The association between variables was analyzed using a t-test, one-way ANOVA, and Chi-square test. The correlation between knowledge, attitude, and behavior scores was conducted using Pearson’s correlation.

## Results

Three hundred forty-five respondents finished the questionnaire; however, 34 respondents were excluded regarding the exclusion criteria described in the methodology. Thus, only 311 overseas students were in the study. They were 55.6% male and 44.4% female, with an average age of 27.5 ± 4.5 years. The respondents came from 23 countries: ASEAN (68.8%) and non-ASEAN (31.2%) countries. Most were studying in non-health science programs (73.3%), with a majority for a master’s degree (57.87%). 55.6% of the students had a monthly allowance of around 429–500 USD (the conversion rate was 1 USD = 35 Baht). Only 14.1% of students had general and dental health insurance, while 22.2% had no health insurance. The characteristics of overseas students are presented in Table 1.

The average scores of knowledge, attitude, and behavior were 8.33 ± 2.84 from 13 (64.1%), 6.33 ± 2.01 from 14 (45.2%), and 5.85 ± 2.04 from 11 (53.2%), respectively. When looking further at the proportion of the respondents with incorrect answers, negative attitudes, and improper behaviors, the results demonstrated that more than 48%, 50%, and 42.5% had knowledge, attitude, and behavior scores below the averages, respectively.

**Table 1:** Characteristics of the study participants

Characteristics	Total (N = 311)	Knowledge Score (13 questions)	p-value*	Attitude Score (14 statements)	p-value*	Behavior Score (11 questions)	p-value*
	N %	Mean SD		Mean SD		Mean SD	
**Gender**			0.927		0.987		0.517
Male	173 55.6	8.32 2.86		6.33 2.05		5.92 2.09	
Female	138 44.4	8.35 2.82		6.33 1.96		5.77 1.97	
**Country**			0.269		0.298		0.327
ASEAN	214 68.8	8.22 3.07		6.41 2.08		5.78 2.0	
Non-ASEAN	97 31.2	8.57 2.23		6.15 1.82		6.02 2.1	
**Level of Study**			0.146		0.204		0.020*
Undergraduate	29 9.3	7.45 3.40		5.73 2.13		6.97 1.86	
Master	180 57.9	8.25 2.81		6.28 2.02		5.70 1.96	
Doctoral	89 28.6	8.65 2.71		6.58 1.91		5.79 2.18	
Postdoctoral	13 4.2	9.23 2.42		6.69 1.97		5.92 1.80	
**Field of Study**			< 0.001*		0.004**		0.604
Health science program	83 26.7	9.55 2.67		6.88 2.18		5.95 2.06	
Non-health science program	228 73.3	7.89 2.77		6.13 1.91		5.82 2.03	
**Monthly Allowance** (USD)			0.516		0.448		0.193
< 285	20 6.4	8.70 3.24		6.05 2.43		6.25 1.55	
285–357	23 7.4	7.83 3.15		6.30 1.86		6.04 1.36	
358–428	20 6.4	7.55 2.37		6.05 1.82		6.25 1.58	
429–500	173 55.6	8.31 2.81		6.23 2.06		5.59 2.00	
501–571	22 7.1	9.09 2.04		6.41 1.59		5.86 2.83	
> 571	53 17.1	8.45 3.07		6.85 1.94		6.32 2.26	
**Health Insurance**			0.262		0.082		< 0.014*
None	69 22.2	8.01 3.25		5.96 2.27		5.48 2.09	
General Health	198 63.7	8.31 2.75		6.35 1.90		5.81 2.06	
General & Oral Health	44 14.1	8.91 2.50		6.82 1.96		6.61 1.64	

*t-test/ANOVA, α < 0.05

**Fig. 1:**
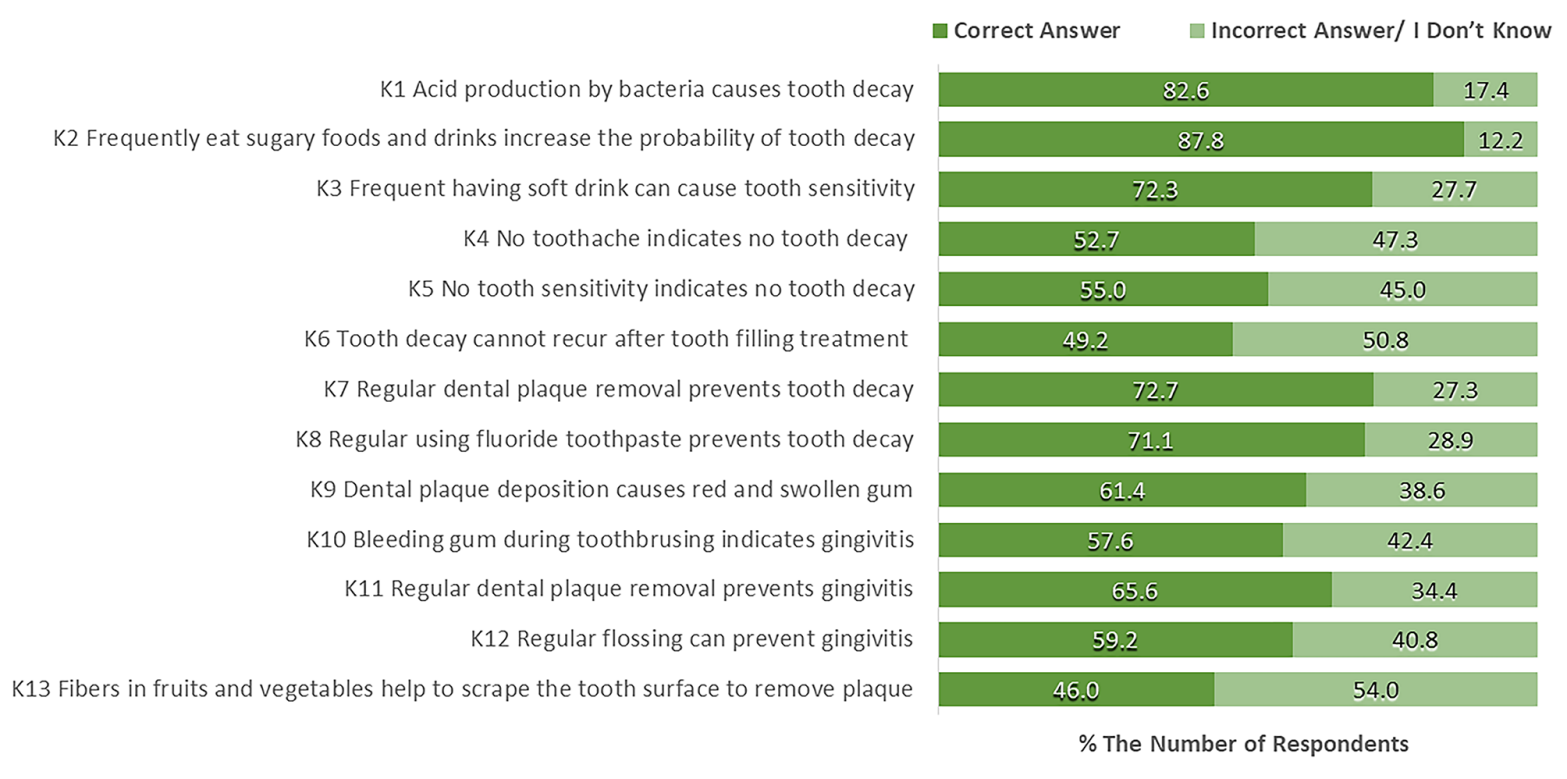
The percentage of overseas university students’ responses to knowledge-related oral health questions (N = 311)

Most of the overseas students had good knowledge about the causes of dental caries (> 80%; K1, K2, K3) and how to prevent dental caries (> 70%; K7, K8). About half of the students did not know about the symptoms of caries and the possibility of recurring dental caries after treatment (K4, K5, K6). For gingivitis, less than those of dental caries, only about 60% of students had the correct knowledge of the cause of gingivitis (K9), how to prevent gingivitis (K11, K12, K13), and misunderstanding that gum bleeding was a physiologic phenomenon (K10; Fig. 1).

**Fig. 2:**
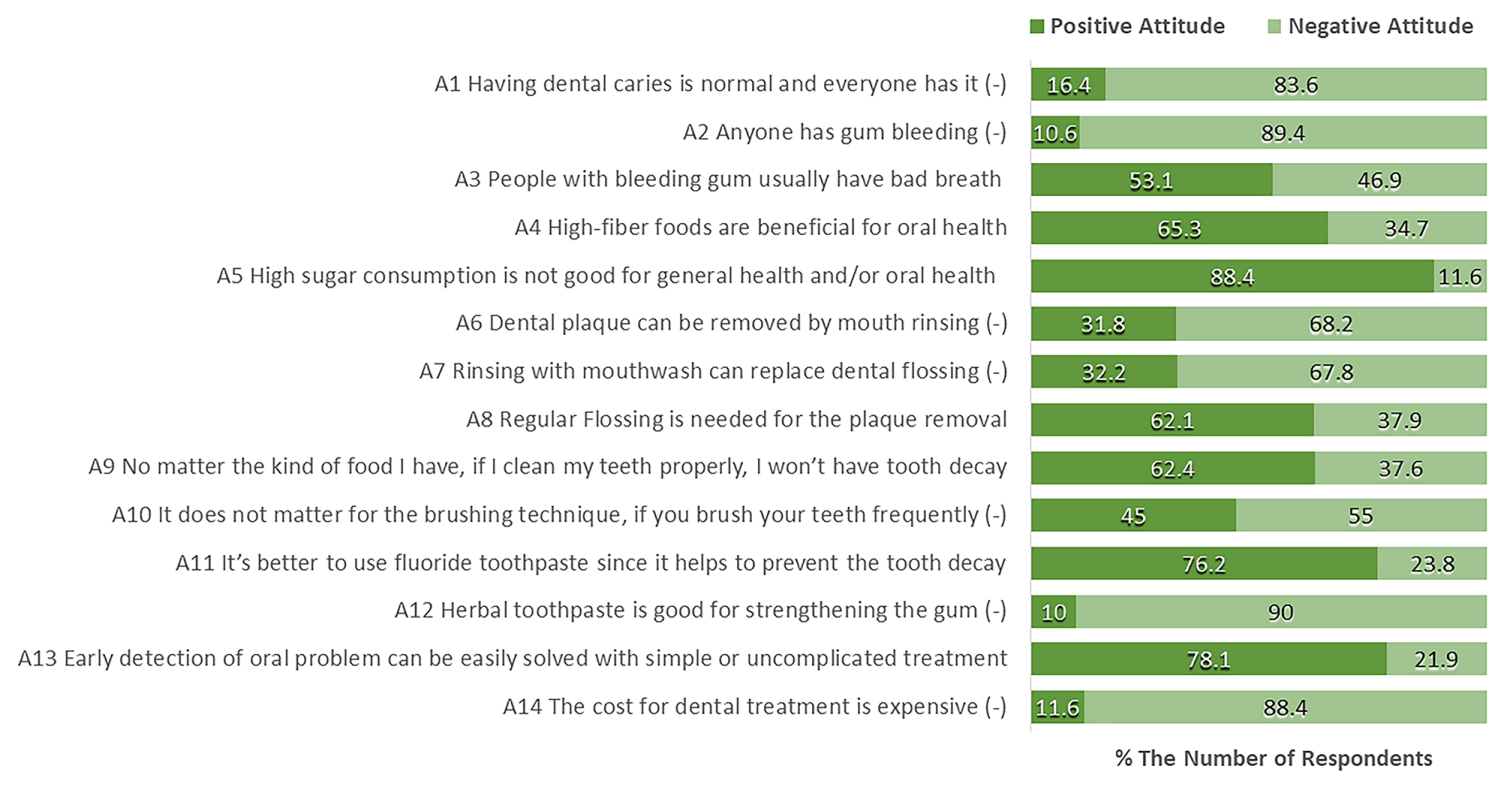
The percentage of overseas university students’ responses to attitude-related oral health statements (N = 311)

The statements for measuring the attitudes were performed with a 5-point Likert scale. For analysis, the answers were grouped into positive and negative attitudes, as shown in Fig. 2. About 90% of the students believed that dental caries and gum bleeding were regular (A1, A2), while some thought gum bleeding related to bad breath (53.1%; A3). Most students agreed that sugary consumption was related to oral health problems (88.4%; A5) and agreed with the benefit of high-fiber foods on tooth self-cleansing (65.3%; A4). About 70% of the students had exemplary attitudes toward oral health prevention, including regular plaque removal by flossing, tooth brushing with fluoride toothpaste, and regular dental visiting (A8, A9, A11, A13); however, paying attention to the brushing technique was essential, as using the wrong technique may cause tooth hypersensitivity and gingival recession (45%; A10). Misunderstanding of the use of mouthwash on plaque removal must be corrected (32%; A6, A7), including herbal toothpaste’s role in strengthening the gum (10%; A12). The hesitance to visit the dentist because of the cost of treatment would cause untreated oral health problems to have serious consequences (11.6%; A14).

**Fig. 3:**
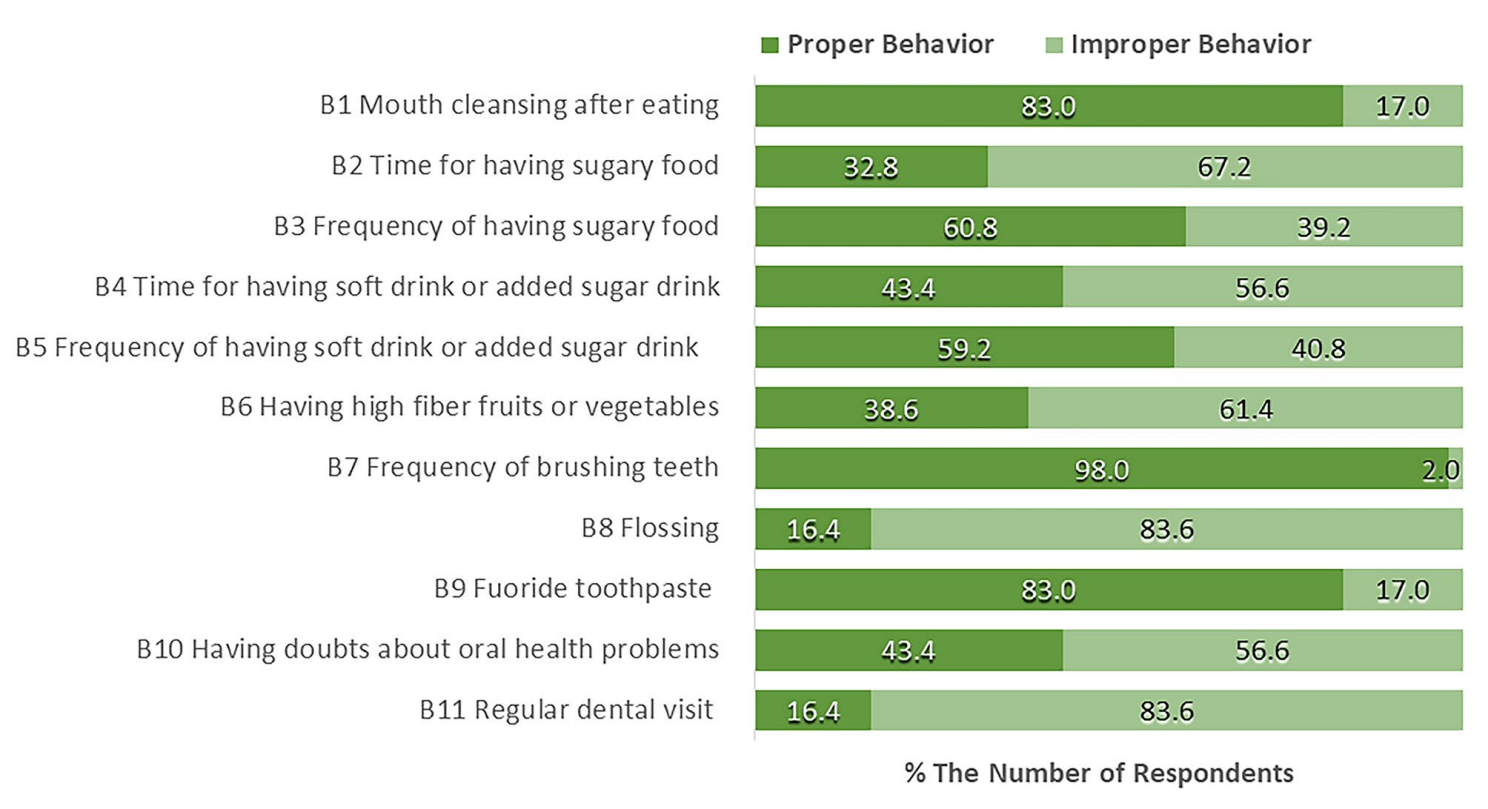
The percentage of overseas university students’ responses on oral health behaviors (N = 311)

Figure 3 shows that around 40% of the students consumed sugar-containing food, desserts, and drinks more often than usual (B3, B5), and around 60% consumed during inappropriate times, such as between meals and before bed (B2, B4). Only a few had regularly high-fiber fruits or vegetables (38.6%; B6). Almost all students cleaned up their oral cavities by either rinsing or toothbrushing after meals with fluoride toothpaste (> 80%; B1, B7, B9). A few students regularly used floss (16.4%; B8). Only 16.4% of the students routinely visited dentists (B11), despite the COVID-19 situation. Meanwhile, during the COVID-19 pandemic, 43.4% of the students preferred to see the dentist if they had oral health problems (B10).

When behaviors were compared before and during the pandemic by the students’ perception, there was a 32.3% increase in the consumption of snacks with added sugar and a 12.6% decrease. Similarly, the consumption of drinks with added sugar has increased by 31.6% and decreased by 13.9%. The frequency of daily toothbrushing has increased by 25.5% but has decreased by 5.8%. It was worth noting that dental flossing has seen a 15% increase in frequency, despite a 10.9% decrease.

Table 1 shows the association of knowledge and attitude with the field of study (p < 0.001 and p = 0.018, respectively), while the behavior was associated with the level of education (p = 0.02) and insurance coverage (p < 0.014). Data was further split by the field of study. After analyses, the result demonstrated an association between knowledge score and the countries only within the non-health science programs (p = 0.013). Meanwhile, it also showed associations of insurance coverage with attitude scores (p = 0.43) and behavior scores (p = 0.32) in the health science program students.

The overall correlation between the knowledge score and behavior score was 0.198 (p < 0.001), while the overall correlation between the attitude score and behavior score was 0.212 (p < 0.001). Further analyses were performed by sub-categorizing the knowledge, attitude, and behavior statements into the diseases’ causes, symptoms, and prevention. There was a positive correlation between knowledge toward behavior regarding the disease symptoms (r = 0.130, p = 0.022). An association was found between knowledge and behavior regarding disease prevention by flossing (Cramer V = 0.170, p = 0.004). A positive correlation existed between attitude toward behavior regarding the plaque deposition-causing disease (r = 0.216, p < 0.001).

**Fig. 4:**
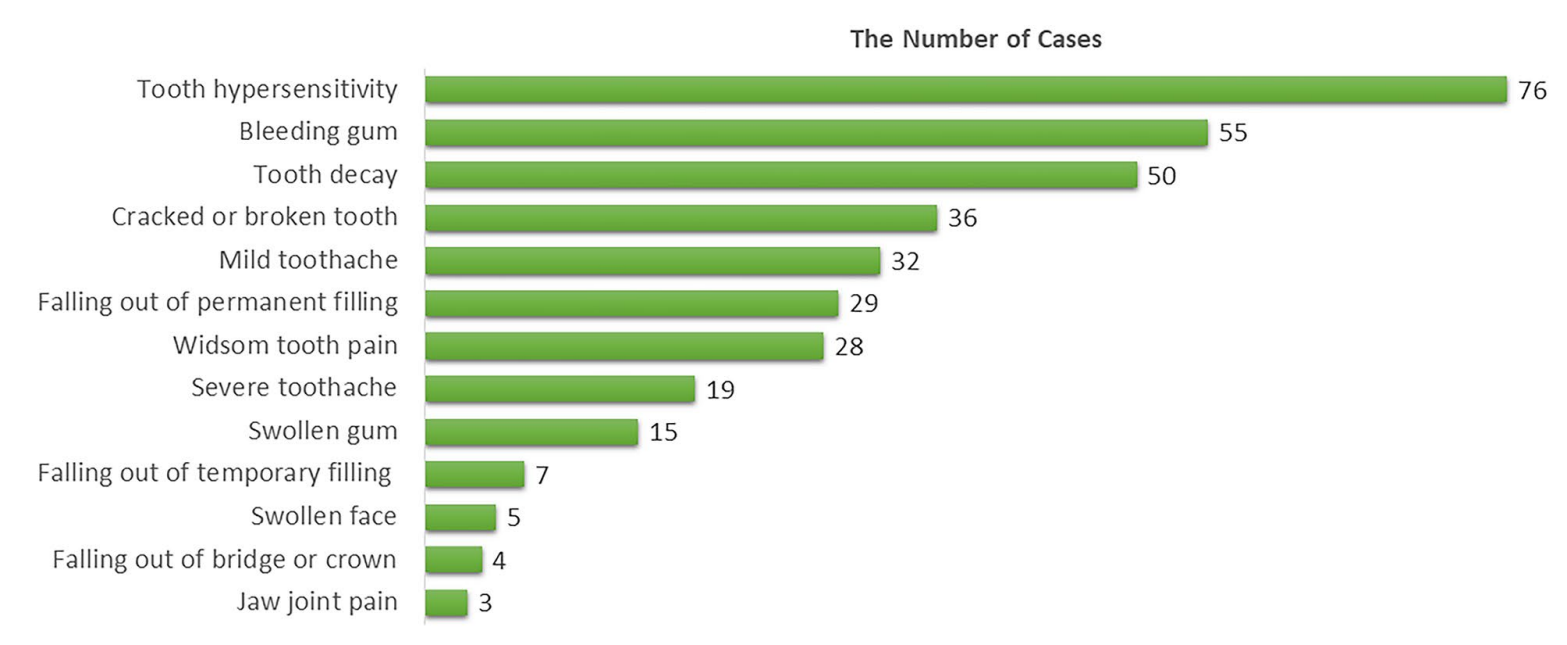
The number of oral health problem cases experienced by overseas university students (number of students = 136; total cases = 359)

**Fig. 5:**
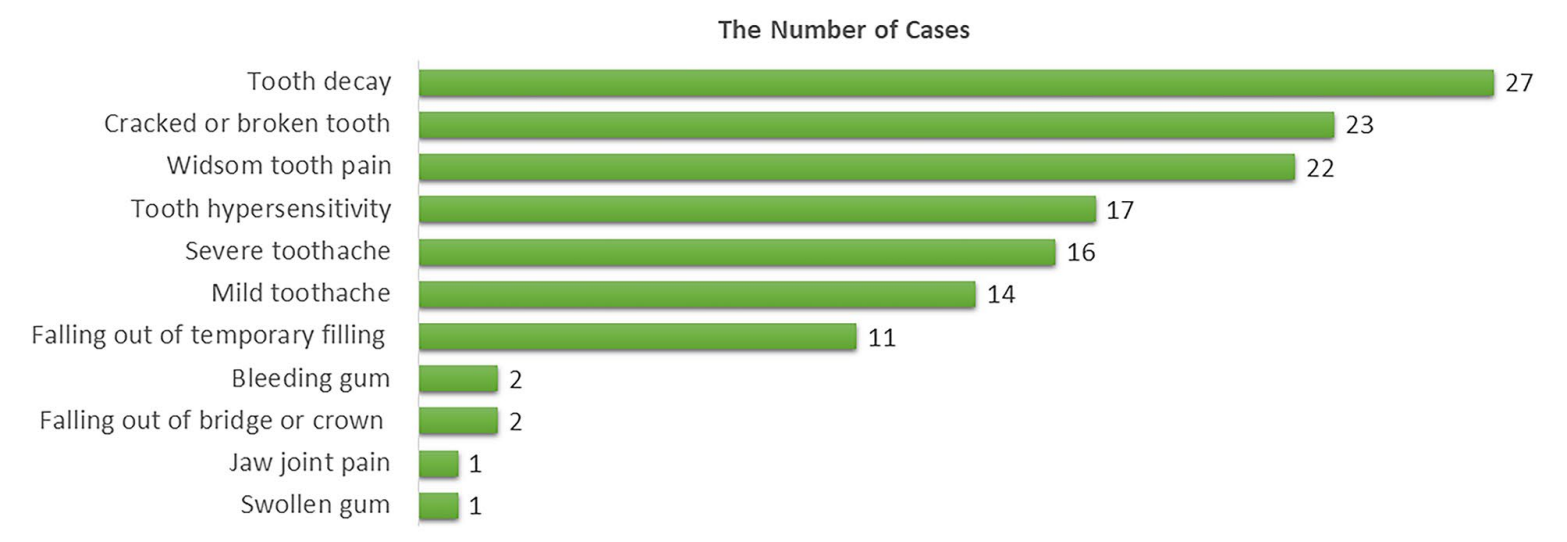
The most concern of oral health problems experienced by overseas university students (number of students = 136; total cases = 359)

During the pandemic, 136 overseas students experienced 359 episodes of oral health problems, which were around 2.64 episodes of oral health problems per person. Details of oral health problems are shown in Fig. 4. Whereas from the student’s point of view, the highly concerned problems differed from the actual problems, as demonstrated in Fig. 5.

The students solved their problems in several ways. Most searched for online information or consulted friends or relatives about relieving pain (62.5%), while a few did an online/hotline consultation with dentists (5.8%). Some went for dental treatments (55.1%), but some could not find open clinics because of the lockdown, or the clinics were not ready for the new standard procedure for infection control (19.8%). Some took medicine (50.7%), while others endured the pain until relieved by themselves (15.4%). Only a few did nothing since there was no disturbance in daily life (5.8%).

We found an association between behavior and oral health problems (p < 0.001). The result showed that the mean behavior score of students with oral health problems (5.37 ± 2.28) was significantly lower than those without problems (6.23 ± 1.74). There was also a negative correlation between the behavior score and oral problem cases (r=-0.204, p < 0.001), meaning overseas students with low oral health behavior scores had more cases of oral health problems.

Interestingly, we also found an association between insurance coverage and dental visits (p = 0.014). 8.7% of the students who had no insurance, 16.2% of the students who were covered by general health insurance, and 29.5% of the students who were covered by general and dental health insurance considered visiting dentists if they doubted their oral health status.

## Discussion

The present study demonstrated that the oral healthcare behavior of overseas university students correlated positively with their knowledge and attitude; however, the level was low. It appeared that many overseas students were not adhering to the recommended protocols for maintaining proper oral hygiene, such as sugary consumption and dental flossing, amidst the challenging circumstances posed by the COVID-19 pandemic. Dental caries and gingivitis were the major oral health problems experienced by overseas students, similar to those in normal situations worldwide. As predictable, there was a negative correlation between behavior and the number of oral health problems. Interestingly, the spectrum of insurance coverage contributed to the decision to make an appointment with a dentist.

In the current study, almost all students had a positive attitude toward sugary intake related to oral health problems. Conversely, over 50% of the students indulge in sugar-laden food and beverages more frequently and at inappropriate times. It is widely accepted that what we eat significantly impacts our health. Unhealthy eating habits are a major contributor to the burden of disease [20]. One of the main culprits is excessive sugar intake which has been linked to various non-communicable diseases, including oral health issues [21]. Recent research revealed that university students have been consuming more sugary foods and drinks since the start of the pandemic, a trend that was observed worldwide [8]. As the students studied online from home, they could easily access sugary treats while attending classes. As a result, their intake of sweets and sugary drinks has increased, and they might even consume these items during study time [22]. Additionally, previous research has shown that students experienced stress increases during the COVID-19 lockdown due to heavy workloads and long hours spent in front of their computers [23, 24]. This stress may also contribute to unhealthy eating habits, such as snacking on sweets and consuming sugary drinks while studying late at night [22]. When living abroad and away from their families, overseas students had the freedom to choose the type of food they like without parental supervision. A systematic review indicated that people who lived alone had a lower diversity of food intake, insufficient essential core nutrition, especially protein, vegetables, and fruits, and a higher likelihood of having an unhealthy dietary pattern [25]. The university’s environment and regulations may hinder efforts to reduce sugar intake on campus, with factors such as time limitations for food availability and strict policies on sugar. As role models, the universities should take steps to address the issue of high sugar consumption and create healthier campus canteens [26]. These findings bring about future interventions to promote healthier habits among universities.

Dental plaque accumulation is a significant risk factor for developing dental caries and periodontal disease. The effective removal of dental plaque is essential for maintaining periodontal health and dental caries prevention. The ADA guideline recommends crucial effective self-care by mechanical control, brushing twice daily with fluoride toothpaste, and flossing once a day. Meanwhile, chemical control is an adjunct measure that may facilitate the removal and prevent the accumulation of microbial plaque [27, 28]. Even though, in normal circumstances, most individuals brush their teeth at least twice a day as recommended, the prevalence of gingivitis remains high in most populations worldwide [17, 28]. Our result demonstrates an increase in the number of times daily toothbrushing during the COVID-19 pandemic. This might be the awareness of oral health, including limited dental clinic access, fear of viral contagiousness by the dental procedure, and hesitation to go out during the lockdown [29]. Also, more than half of the respondents believed the brushing technique was unimportant if they brushed their teeth more frequently. Wrong brushing techniques are considered harmful because vigorous scrubbing can lead to gingival recession, and abrasives in toothpaste can cause tooth abrasions, which leads to tooth hypersensitivity. Modified Bass’ method is the recommended tooth brushing technique [30, 31].

Regarding attitude, most students believed using herbal toothpaste strengthens the gum, although 83% used fluoride toothpaste. Herbal extracts have increased attraction because they are natural, not chemical, giving a sense of safety, though a plurality of effects in herbal extracts makes their action non-specific. Moreover, there were periodic reports of allergy or hypersensitivity reactions [32, 33]. The meta-analyses revealed that fluoride toothpaste was more efficient in reducing dental plaque than herbal, and non-herbal and non-fluoride toothpaste, which appear equally effective [34]. One of the shared potential mechanisms of action of herbal toothpaste is that their active ingredients can penetrate biofilm and prevent plaque accumulation, therefore preventing bacterial colonization on the tooth surfaces [35]. Mouth rinse is an adjunct for dental plaque control. A meta-analysis found that chlorhexidine was superior to essential oil mouth rinses in terms of plaque reduction, though with the adverse effect in long-term use, while there were no significant differences in reducing gingivitis [34]. Hence, there were low recommendations for using herbal preparations to substitute conventional oral hygiene products [36]. A recent meta-analysis showed that flossing, in addition to toothbrushing, reduced gingivitis compared to toothbrushing alone [37, 38]. However, there was not enough evidence to support the effectiveness of flossing in preventing dental caries [39]. The study by Broadbent JM. and colleagues demonstrated that 49% of adolescents thought flossing was unimportant, whereas almost all routinely brushed [40]. Our result showed a similar direction, with only 16% flossing.

Compared to non-health science programs, overseas students who studied in health science programs had higher knowledge scores and more positive attitudes, similar to previous studies [40–43]. It is worth noting that having a background in health science provided a better understanding of general health, including oral health; however, it was not a significant difference in behavior between these two groups. In the present study, behavior was, instead, associated with the level of education and insurance coverage. Our research revealed a low but positive correlation between knowledge toward behavior, and attitude toward behavior. Theoretically, possessing knowledge or attitude should lead to appropriate behavior. However, the positive correlation outcomes of similar studies varied depending on factors such as sample size, region, duration, and when the research was performed [41]. Several studies suggested that knowledge must be given as early as possible to promote the development and retention of positive attitude and good behavior, which needed several steps and took time [44, 45]; however, various factors could influence the outcome. People’s oral health attitudes are not necessarily fixed. One study reported that a substantial proportion of the population was likely to change their beliefs about oral health practices between adolescence and young adulthood [40]. Therefore, it is necessary to implement interventions that encourage sustainable positive attitudes towards oral behavior. As our focus was on overseas students, the low positive correlation may be attributed to unmeasured socio-cultural environment factors such as peer influence, inconvenient living arrangements, and high cost of living.

Education is an essential tool for maintaining oral health [41]. Our result indicated that the field of study affected overseas students’ knowledge and attitudes. To implement and retain proper knowledge and attitudes, the universities may provide free elective courses in basic health to improve health literacy, especially for non-health science program students. In addition, undergraduate students had significantly higher mean behavior scores than master, doctoral, and postdoctoral students. These latter groups of students might be affected by workloads regarding their study levels which might cause them to neglect themselves unintentionally [46]. Further analyses were performed for an association between behavior scores and oral health problems in overseas students. Our result, along with previous studies showed that students who had appropriate oral health behavior had a lower number of oral health problems [47]. About 43.7% of the overseas students in the current study had at least one episode of oral health problem.

Besides clinic closing during the lockdown period, it was concerning that 60% of overseas students hesitated to visit dentists due to COVID-19 infection risks associated with dental procedures. This fear could prevent them from addressing any oral health issues they may have [11]. Some students turned to online searches and consulted friends for advice, but it was essential to be cautious as there was a risk of misinformation [48]. Students may benefit from using a health web engine instead of a general search engine like Google to reduce this risk [48, 49]. Despite the potential for false or misleading information, this trend showed that overseas students were taking proactive measures to safeguard their health. Consulting with dentists must be the best; however, before the pandemic, telehealth or tele-dentistry had the primary purpose for remote healthcare [50, 51]. Not only overseas students but most people in urban areas were unfamiliar with tele-dentistry [50]. Thus, it was no surprise that few students chose this method to solve their problems. After the pandemic, tele-dentistry may be added into everyday practice for the benefit of decongesting clinics, improving patient flow, and reducing travel and associated costs for patients [52]. A barrier to implementation is difficulty providing accurate diagnoses based on videos or static images without necessarily diagnostic maneuvers such as percussion, palpation, and radiographic investigation [51, 53]. Regarding the limitation of telediagnosis, some aspects of tele-dentistry may be applied. Tele-triage prioritizes patients needing urgent care, nonprocedural care, hygiene assessments, specialist consultations, and telemonitoring can follow up on treatments [52].

Living expense is a major consideration for overseas students. Healthcare is one of the expenses that must be worried about, which possibly causes the students to forego dental treatment even if needed. Based on our data, students with both general and dental health insurance had higher behavior mean scores than those with general health insurance alone. The coverage of health insurance was associated with dental visits (p = 0.014). The previous reports suggested that dental insurance was associated with improved dental visiting behaviors and oral health status outcomes regarding the affordability of dental procedures [54, 55]; thus, health insurance schemes should include regular dental check-ups to emphasize prevention-oriented dental care. Language can also be a barrier for overseas students seeking dental care. Misunderstandings can arise, making it difficult for students to feel satisfied with the treatments [56].

To effectively address these issues, First-year students should receive comprehensive welfare support from the universities, including oral health check-ups and early lesion detection screenings, to raise their awareness and encourage them to prioritize their oral health care needs. Moreover, tele-dentistry can support universities to maintain students’ oral health and save on dental visit expenses by using teleconsultation, triage, and monitoring, regardless of students’ location.

Initial conclusions can be drawn as the study had limitations. Recall bias was a critical limitation; however, it might be reduced since the pandemic was life-changing. The participants were more likely to recall what occurred at two different time points than during the normal situation. The study was a cross-sectional survey conducted via an online Google form using a convenient sampling method; therefore, an even distribution of the sample population among all faculties could not be avoided. All information was assessed by self-report, which may have biased the results. This study only collected data from Chulalongkorn University students. Further study on a larger scale of the overseas student population must be conducted to develop supportive oral healthcare policies for this student group.

## Conclusion

This study demonstrated that oral healthcare habits of overseas university students positively correlated with knowledge and attitude. Dental caries and gingivitis continued to be the most common oral health issues among these students. A negative correlation was found between behavior and the number of oral health problems experienced. Furthermore, the field of study impacted the knowledge and attitude of overseas students toward oral health. It was also important to note that insurance coverage affected decisions to seek dental care.

## Data Availability

The datasets generated and/or analyzed during the current study are not publicly available due the data protection guidelines according to the ethics approval but are available from the corresponding author on reasonable request.

## References

[1] Platto S, Wang Y, Zhou J, Carafoli E (2021). History of the COVID-19 pandemic: origin, explosion, worldwide spreading. Biochem Biophys Res Commun.

[2] Goodell JW (2020). COVID-19 and finance: agendas for future research. Financ Res Lett.

[3] Dhawan S (2020). Online learning: a panacea in the Time of COVID-19 Crisis. J Educ Tech Sys.

[4] Suphanchaimat R, Teekasap P, Nittayasoot N, Phaiyarom M, Cetthakrikul N (2022). Forecasted Trends of the New COVID-19 Epidemic due to the Omicron variant in Thailand, 2022. Vaccines.

[5] Djalante R, Nurhidayah L, Van Minh H, Phuong NTN, Mahendradhata Y, Trias A (2020). COVID-19 and ASEAN responses: comparative policy analysis. Prog Disaster Sci.

[6] Palmer K, Bschaden A, Stroebele-Benschop N (2021). Changes in lifestyle, diet, and body weight during the first COVID-19 ‘lockdown’ in a student sample. Appetite.

[7] López-Moreno M, López MTI, Miguel M, Garcés-Rimón M (2020). Physical and psychological Effects related to Food Habits and Lifestyle Changes Derived from COVID-19 Home Confinement in the Spanish Population. Nutrients.

[8] Bennett G, Young E, Butler I, Coe S (2021). The impact of Lockdown during the COVID-19 outbreak on Dietary Habits in various Population Groups: a scoping review. Front Nutr.

[9] Dar-Odeh N, Babkair H, Abu-Hammad S, Borzangy S, Abu-Hammad A, Abu-Hammad O (2020). COVID-19: Present and Future Challenges for Dental Practice. Int J Environ Res Pub Health.

[10] Banakar M, Bagheri Lankarani K, Jafarpour D, Moayedi S, Banakar MH, Mohammad Sadeghi A (2020). COVID-19 transmission risk and protective protocols in dentistry: a systematic review. BMC Oral Health.

[11] Zhang S, Liu C, Zhang C, Jiang H, Tai B, Du M (2021). Impact of COVID-19 on the oral health of adults in Wuhan and China: results of a nationwide online cross-sectional questionnaire survey. BMC Oral Health.

[12] Ministry of Higher Education, Science, Research and Innovation of Thailand. International Student Mobility Statistics. http://info.mhesi.go.th/info/ accessed 18 August 2023.

[13] Winkelmann J, Gómez Rossi J, Schwendicke F, Dimova A, Atanasova E, Habicht T (2022). Exploring variation of coverage and access to dental care for adults in 11 european countries: a vignette approach. BMC Oral Health.

[14] Mathrani A, Sarvesh T, Umer R (2021). Digital divide framework: online learning in developing countries during the COVID-19 lockdown. Global Soc Educ.

[15] Bonsaksen T, Chiu V, Leung J, Schoultz M, Thygesen H, Price D (2022). Students’ Mental Health, Well-Being, and loneliness during the COVID-19 pandemic: a cross-national study. Healthcare.

[16] Yuan T, Li XD, Zhang M, Tao XB, Xu SJ, Liu H (2022). Impact of the eHealth literacy, knowledge and attitudes on COVID-19 prevention behavior among residents in the second year of the COVID-19 pandemic: a cross-sectional study in Anhui Province, China. Front Pub Health.

[17] Wen PYF, Chen MX, Zhong YJ, Dong QQ, Wong HM (2022). Global Burden and Inequality of Dental Caries, 1990 to 2019. J Dent Res.

[18] Uakarn C, Chaokromthong K, Sintao N (2021). Sample size estimation using Yamane and Cochranand Krejcie and Morgan and Green Formulas and Cohen Statistical Power Analysis by G*Power and comparisons. Apheit Inter J.

[19] Stratton SJ (2021). Population Research: convenience sampling strategies. Prehosp and Disaster Med.

[20] Qiao J, Lin X, Wu Y, Huang X, Pan X, Xu J (2021). Global burden of non-communicable diseases attributable to dietary risks in 1990–2019. J Hum Nutr Diet.

[21] Moynihan P (2016). Sugars and Dental Caries: evidence for setting a recommended threshold for Intake. Adv Nutr.

[22] Elsalem L, Al-Azzam N, Jum’ah AA, Obeidat N, Sindiani AM, Kheirallah KA (2020). Stress and behavioral changes with remote E-exams during the Covid-19 pandemic: a cross-sectional study among undergraduates of medical sciences. Ann Med Surg.

[23] Ubolnuar N, Luangpon N, Pitchayadejanant K, Kiatkulanusorn S (2022). Psychosocial and physical predictors of stress in University students during the COVID-19 pandemic: an observational study. Healthcare.

[24] Kita Y, Yasuda S, Gherghel C (2022). Online education and the mental health of faculty during the COVID-19 pandemic in Japan. Sci Rep.

[25] Hanna KL, Collins PF (2015). Relationship between living alone and food and nutrient intake. Nutr Rev.

[26] Roy R, Soo D, Conroy D, Wall CR, Swinburn B (2019). Exploring University Food Environment and On-Campus Food Purchasing Behaviors, Preferences, and opinions. J Nutr Educ Behav.

[27] Selwitz RH, Ismail AI, Pitts NB (2007). Dental caries. The Lancet.

[28] Lee Y (2013). Diagnosis and Prevention Strategies for Dental Caries. J Lifestyle Med.

[29] Wdowiak-Szymanik A, Wdowiak A, Szymanik P, Grocholewicz K, Pandemic COVID-19 Influence on Adult’s Oral Hygiene, Dietary Habits and Caries Disease - Literature Review. Int J Environ Res Public Health. 10.3390/ijerph191912744 PMC9566618 36232043

[30] Janakiram C, Varghese N, Venkitachalam R, Joseph J, Vineetha K. Comparison of modified Bass, Fones and normal tooth brushing technique for the efficacy of plaque control in young adults- A randomized clinical trial. J Clin Exp Dent. 2020;12(2):e123-9. 10.4317/jced.55747 PMC7018473 32071693

[31] Ali AT, Varghese S, Shenoy R (2022). Association between cervical abrasion, oral hygiene practices and buccolingual dimension of tooth surfaces: a cross-sectional study. J Pharm Bioallied Sci.

[32] Joshi C, Shukla P (2015). Plasma cell gingivitis. J Indian Soc Periodontol.

[33] Anil S (2007). Plasma cell gingivitis among herbal toothpaste users: a report of three cases. J Contemp Dent Pract.

[34] Janakiram C, Venkitachalam R, Fontelo P, Iafolla TJ, Dye BA (2020). Effectiveness of herbal oral care products in reducing dental plaque & gingivitis - a systematic review and meta-analysis. BMC Complement Med Ther.

[35] Wu-Yuan CD, Green L, Birch WX (1990). In vitro screening of chinese Medicinal Toothpastes: their Effects on Growth and Plaque formation of Mutans Streptococci. Caries Res.

[36] Van Leeuwen MPC, Slot DE, Van der Weijden GA (2011). Essential oils compared to Chlorhexidine with respect to Plaque and Parameters of Gingival inflammation: a systematic review. J Periodontol.

[37] Milleman J, Bosma ML, McGuire JA, Sunkara A, McAdoo K, DelSasso A (2022). Comparative effectiveness of Toothbrushing, Flossing and Mouthrinse Regimens on Plaque and Gingivitis: a 12-week virtually supervised clinical trial. J Dent Hyg.

[38] Edlund P, Bertl K, Pandis N, Stavropoulos A (2022). Efficacy of power-driven interdental cleaning tools: a systematic review and meta-analysis. Clin Exp Dent Res.

[39] Amarasena N, Gnanamanickam ES, Miller J (2019). Effects of interdental cleaning devices in preventing dental caries and periodontal diseases: a scoping review. Aust Dent J.

[40] Broadbent JM, Thomson WM, Poulton R (2016). Oral health beliefs in adolescence and oral health in Young Adulthood. J Dent Res.

[41] Zheng S, Zhao L, Ju N, Hua T, Zhang S, Liao S (2021). Relationship between oral health-related knowledge, attitudes, practice, self-rated oral health and oral health-related quality of life among chinese college students: a structural equation modeling approach. BMC Oral Health.

[42] Sharda AJ, Shetty S (2010). A comparative study of oral health knowledge, attitude and behaviour of non-medical, para-medical and medical students in Udaipur city, Rajasthan, India. Int J Dent Hyg.

[43] Farsi NJ, Merdad Y, Mirdad M, Batweel O, Badri R, Alrefai H (2020). Oral health knowledge, attitudes, and Behaviors among University students in Jeddah, Saudi Arabia. Clin Cosmet Invest Dent.

[44] Herlitz L, MacIntyre H, Osborn T, Bonell C (2020). The sustainability of public health interventions in schools: a systematic review. Implement Sci.

[45] Gardner B, Rebar AL, D D (2019). Habit formation and behavior change. Oxford research encyclopedia of psychology.

[46] Ahalli S, Fort E, Bridai Y, Baborier N, Charbotel B (2022). Mental health and working constraints of first-year PhD students in health and science in a french university: a cross-sectional study in the context of occupational health monitoring. BMJ Open.

[47] Tadin A, Poljak Guberina R, Domazet J, Gavic L (2022). Oral Hygiene Practices and oral health knowledge among students in Split, Croatia. Healthcare.

[48] Di Sotto S, Viviani M (2022). Health Misinformation detection in the Social web: an overview and a Data Science Approach. Int J Environ Res Pub Health.

[49] Darwish O, Tashtoush Y, Bashayreh A, Alomar A, Alkhaza’leh S, Darweesh D (2023). A survey of uncover misleading and cyberbullying on social media for public health. Cluster Comput.

[50] Yaembut N, Pochanuk N, Soparat P, Sukhumalind P (2021). Teledentistry for development of dental health service system. Thai J Health Prom Environ Health.

[51] Mahdavi A, Atlasi R, Naemi R (2022). Teledentistry during COVID-19 pandemic: scientometric and content analysis approach. BMC Health Serv Res.

[52] Farzandipour M, Nabovati E, Sharif R (2023). The effectiveness of tele-triage during the COVID-19 pandemic: a systematic review and narrative synthesis. J Telemed Telecare.

[53] Cheuk R, Adeniyi A, Farmer J, Singhal S, Jessani A (2023). Teledentistry use during the COVID-19 pandemic: perceptions and practices of Ontario dentists. BMC Oral Health.

[54] Zivkovic N, Aldossri M, Gomaa N, Farmer JW, Singhal S, Quiñonez C (2020). Providing dental insurance can positively impact oral health outcomes in Ontario. BMC Health Serv Res.

[55] Bayat F, Vehkalahti MM, Zafarmand AH, Tala H (2008). Impact of insurance scheme on adults’ dental check-ups in a developing oral health care system. Eur J Dent.

[56] Imafuku R, Nagatani Y, Shoji M (2022). Communication management processes of dentists providing Healthcare for Migrants with Limited Japanese Proficiency. Int J Environ Res Pub Health.

